# VIP-Expressing GABAergic Neurons: Disinhibitory vs. Inhibitory Motif and Its Role in Communication Across Neocortical Areas

**DOI:** 10.3389/fncel.2022.811484

**Published:** 2022-02-10

**Authors:** Alfonso junior Apicella, Ivan Marchionni

**Affiliations:** ^1^Department of Biology, Neurosciences Institute, University of Texas at San Antonio, San Antonio, TX, United States; ^2^Department of Biomedical Sciences, University of Padova, Padua, Italy; ^3^Padova Neuroscience Center (PNC), University of Padova, Padua, Italy

**Keywords:** GABAergic neurons, vasoactive intestinal polypeptide, cortex, inhibition, disinhibition, local circuit, long-range axons

## Abstract

GABAergic neurons play a crucial role in shaping cortical activity. Even though GABAergic neurons constitute a small fraction of cortical neurons, their peculiar morphology and functional properties make them an intriguing and challenging task to study. Here, we review the basic anatomical features, the circuit properties, and the possible role in the relevant behavioral task of a subclass of GABAergic neurons that express vasoactive intestinal polypeptide (VIP). These studies were performed using transgenic mice in which the VIP-expressing neurons can be recognized using fluorescent proteins and optogenetic manipulation to control (or regulate) their electrical activity. Cortical VIP-expressing neurons are more abundant in superficial cortical layers than other cortical layers, where they are mainly studied. Optogenetic and paired recordings performed in *ex vivo* cortical preparations show that VIP-expressing neurons mainly exert their inhibitory effect onto somatostatin-expressing (SOM) inhibitory neurons, leading to a disinhibitory effect onto excitatory pyramidal neurons. However, this subclass of GABAergic neurons also releases neurotransmitters onto other GABAergic and non-GABAergic neurons, suggesting other possible circuit roles than a disinhibitory effect. The heterogeneity of VIP-expressing neurons also suggests their involvement and recruitment during different functions *via* the inhibition/disinhibition of GABAergic and non-GABAergic neurons locally and distally, depending on the specific local circuit in which they are embedded, with potential effects on the behavioral states of the animal. Although VIP-expressing neurons represent only a tiny fraction of GABAergic inhibitory neurons in the cortex, these neurons’ selective activation/inactivation could produce a relevant behavioral effect in the animal. Regardless of the increasing finding and discoveries on this subclass of GABAergic neurons, there is still a lot of missing information, and more studies should be done to unveil their role at the circuit and behavior level in different cortical layers and across different neocortical areas.

## Introduction

The cortical processing is characterized by the interaction of excitatory/glutamatergic pyramidal neurons and the inhibitory/GABAergic neurons. Every cortical activity is shaped by two opposing and inseparable events: synaptic excitation and inhibition. In addition, these two opposing forces, together with their strength and temporal relationship, control the cortex’s function in space and time.

The cortex contains about 70–80% of pyramidal neurons, while the remaining 20–30% is represented by a different population of GABA-releasing neurons (for review see Feldmeyer, 2012). Despite being a small fraction of the entire neocortical population, these GABAergic neurons are a very heterogeneous class given their anatomical, electrophysiological, and molecular properties.

Over the past 100 years, scientists have debated the classification and nomenclature of neurons. Despite all the effort, a satisfactory consensus has not been reached yet. This is particularly true for the cortical GABAergic neurons due to the enormous amount of morphological, molecular, and physiological data that has been accumulated on these neurons over the past two decades (for review see DeFelipe et al., 2013). In 2008, the Petilla Interneuron Nomenclature Group (PING) divided cortical GABAergic neurons based on the expression of specific molecular markers into five main groups: the parvalbumin (PV)-expressing neurons, including chandelier and basket GABAergic neurons, the Somatostatin (SOM)-expressing, such as Martinotti cells, the neuropeptide Y (NPY)-expressing but not SOM-expressing neurons, vasoactive intestinal polypeptide (VIP)-expressing neurons, and cholecystokinin (CCK)-expressing but not SOM-or VIP-expressing neurons (Petilla Interneuron Nomenclature Group, Ascoli et al., 2008). A more recent study from Tasic et al. (2016), using single-cell RNA sequencing (scRNAseq) and statistical clustering, identified 23 transcriptomic types of GABAergic neurons in the mouse visual cortex. In a subsequent study, Tasic (2018)∼60 types of GABAergic neurons conserved between the visual and frontal cortex were identified. However, the major subclasses, types, and their hierarchical relationships are broadly consistent with previous studies in which the cortical GABAergic neurons can be subdivided into three non-overlapping subclasses of neurons: (1) Parvalbumin (PV)—expressing neurons, Somatostatin (SOM)—expressing neurons, and 5-hydroxytryptamine, or ionotropic serotonin receptor (5HT3aR)-expressing neurons (Rudy et al., 2011; Markram et al., 2015; Tremblay et al., 2016).

Based on the characteristic anatomical and wiring diagrams, the canonical circuit diagram has determined the following synaptic pattern between pyramidal and GABAergic neurons:

(1) The PV-expressing neurons, representing about 30–50% of all cortical GABAergic neurons, preferentially target the peri-somatic region of the pyramidal neurons, allowing these neurons to quickly control the spike output of the pyramidal neurons.
(2) The SOM-expressing neurons comprise approximately 30% of all cortical GABAergic neurons. These neurons target the dendrites of pyramidal neurons preferentially in order to control their distal inputs.
(3) Within the main three sub-classes of GABAergic neurons in the cortex, the Vasoactive Intestinal Polypeptide (VIP)-expressing neurons represent about 40% of the ionotropic serotonin receptor (5HT3aR) neurons. Recent studies have shown that their activity inhibits both PV-expressing neurons and SOM-expressing neurons, thereby controlling the activity of pyramidal neurons through the other two classes of GABAergic neurons (Rudy et al., 2011; Tremblay et al., 2016; Huang and Paul, 2019).

Cortical and subcortical brain structures are anatomically and functionally interconnected by long-distance axons. The dynamic interaction between different brain areas is a key component of the brain function that serves as the “decoding” and “coding” system to interpret the external world and influence our behavior. Then, local neuronal circuits within each brain area receive the stream of information from the distal region, process, and pass the processed information to downstream brain areas. Different excitatory and inhibitory cortical circuits control and modulate postsynaptic neuron activity. GABAergic inhibitory neurons directly inhibit excitatory neurons and make synaptic contacts with other GABAergic neurons, therefore producing a disinhibitory outcome on pyramidal neurons. Initially, the disinhibitory circuit motif involving VIP-expressing neurons controlling local microcircuits by preferentially targeting other GABAergic neurons was identified in the hippocampus (Acsady et al., 1996; Hajos et al., 1996). Since the initial discovery of this VIP-expressing neuron disinhibitory motif, numerous studies have focused their attention aimed to understand the neuronal connectivity (morpho-functional mapping of VIP-expressing neurons) of these neurons in the hippocampus (Chamberland et al., 2010; Donato et al., 2013; Tyan et al., 2014; Francavilla et al., 2018).

Here we address the role of VIP-expressing neurons in the mouse neocortex by first summarizing the recent anatomical and synaptic connectivity of this canonical disinhibitory motif and its function in behaving mice. Then, we will discuss alternative mechanisms complementary to the disinhibitory motif. Finally, we will discuss a new subclass of neocortical VIP-expressing neurons characterized by long-range GABAergic projection and their potential role in routing information across brain areas *via* an inhibitory and/or disinhibitory motif in healthy and diseased brains.

## Anatomical, Electrophysiological, and Molecular Properties of Cortical VIP-Expressing Neurons

Neuronal anatomy is strictly correlated to the neuron’s function. The somato-dendritic compartments and the axonal arbor indicate how the specific neuron receives its input and routes its output within the microcircuit and across different brain structures.

VIP-expressing neurons constitute only 1–2% of the entire cortical population. In general, these neurons span all the cortical layers even though they are more present in the supra-granular layers (layer 2/3), representing the most abundant subclass of GABAergic neurons (Pronneke et al., 2015, 2020; Zhou et al., 2017; Almasi et al., 2019; Guet-McCreight et al., 2020; Badrinarayanan et al., 2021). At the level of the supragranular layers (1 and 2/3), the VIP-expressing neurons share a similar, bipolar dendritic morphological pattern characterized by a dendritic arborization confined in the supra-granular layers reaching layer 1. On the other side, its organization becomes more heterogeneous in the deeper layers (4, 5, and 6) of the cortex, where additional studies revealed different dendritic morphology such as (1) multipolar single tufted, (2) bi-tufted, and (3) horizontally bipolar with dendrites that span all cortical layers (Vucurovic et al., 2010; Pronneke et al., 2015, 2020; Sohn et al., 2016; Emmenegger et al., 2018).

The morphological diversity of the dendritic arbors of the VIP-expressing neurons indicates that this type of neuron may be recruited by different intra- and inter-layer local inputs and from long-range inputs coming from different brain regions providing layer-specific inhibition. The local vs. long-range recruitment of the VIP-expressing neurons could influence, in principle, different local circuits of the cortex and might have an essential role in specific tasks during different behavioral states of the animal (Cirelli and Tononi, 2000).

Another interesting aspect of the morphological properties of the VIP-expressing neurons is that the axon often starts at dendrites oriented toward the white matter rather than the soma (Pronneke et al., 2015), giving a characteristic feature to this class of neurons and possibly an enhanced chance to elicit action potentials at lower activation threshold (Thome et al., 2014). In addition, in different cortical areas, the axons of VIP-expressing neurons in the superficial layers extend vertically, covering all cortical layers without spreading horizontally (Vucurovic et al., 2010; Pronneke et al., 2015; Karnani et al., 2016b; Badrinarayanan et al., 2021). On the other hand, VIP-expressing neurons in deeper layers in the barrel cortex are mainly local, with some branches entering the white matter (Pronneke et al., 2015).

All VIP-expressing neurons have symmetric type II synapses, inhibitory, predominately located onto the dendrites, but a small percentage target spine and somata of the postsynaptic neurons (Zhou et al., 2017). VIP-expressing neurons do not contact the somata but only the dendrites of the layer 1 GABAergic neurons. Only layer 6 VIP-expressing neurons have a higher proportion contacting the somata of the postsynaptic neurons. Each VIP-expressing neuron of the S1 cortex makes one single bouton on the postsynaptic neuron. In contrast, only a small fraction of postsynaptic neurons is targeted with two or three synaptic boutons (Zhou et al., 2017). In layer 2/3, VIP-expressing neurons of the somatosensory cortex (S1) are homogeneously distributed within columns and septum, but in deeper layers are mainly present in the septal (Almasi et al., 2019). According to their electrophysiological properties, VIP-expressing neurons are characterized by heterogeneous firing properties along with the cortical layers with different proportions of the four firing types. The major firing patterns found in the VIP-expressing neurons are: (1) irregular spiking, (2) continuous adapting, (3) burst spiking, and (4) a low percentage high threshold non-adapting, especially in the deep layer (Pronneke et al., 2015, 2020). This wide heterogeneity of firing patterns suggests different biophysical membrane features within the subclass of VIP-expressing neurons. The irregular spiking VIP-expressing neurons show a non-homogeneous firing pattern during depolarizing current at the threshold. Particularly, Porter et al. (1998) suggested that in the rat motor cortex, the slowly inactivating I_*D*_-like K current is involved in generating the irregular discharge pattern of the VIP-expressing neurons. Indeed, about 20% of the VIP-expressing neurons in the superficial layer of the barrel cortex display burst spiking activity due to the expression of T-type calcium and HCN channels (Bean, 2007). It was also shown that the burst spiking activity recorded at the resting membrane potential turns into a regular firing depending on the membrane potentials and the activation of nicotinic receptors (by the acetylcholine) and serotonin (5HT) ionotropic and metabotropic receptors. The latest firing pattern was not found in the deeper layers (4–6) (Pronneke et al., 2020). The most abundant firing pattern in the superficial and deep layer is the continuous adapting. The expression of calcium-activated (KCa; both BK and SK) potassium channels are the critical key mediators to control this spike frequency during the sustained suprathreshold depolarized current. These variabilities found in this subclass of GABAergic neurons suggest that the timing and modality of the release of the inhibitory neurotransmitter is layer and subtype-specific.

Moreover, the firing patterns show no correlation with neurons’ morphological properties, making the classification more complex. In addition to the very heterogeneous properties of the firing patterns, the VIP-expressing neurons are also characterized by a large diversity of membrane properties in response to hyperpolarizing current and near rheobase (Pronneke et al., 2015). Different membrane properties are found both in the barrel cortex (Pronneke et al., 2015, 2020) and in the medial entorhinal cortex (MEC) between the supragranular and infragranular cortex (Badrinarayanan et al., 2021), suggesting distinct physiological roles of these neurons.

Interestingly a subclass of VIP-expressing neurons expresses choline acetyltransferase (ChAT) at the presynaptic terminal (Bayraktar et al., 1997; Porter et al., 1998) and showed undistinguished intrinsic properties with the ChAT negative VIP-expressing neurons, even though this subpopulation is less excitable and has lower input resistance (Obermayer et al., 2019; Dudai et al., 2020; Granger et al., 2020).

Although VIP-expressing neurons are a specific subclass of GABAergic neurons that do not overlap with the two major classes, VIP can be co-expressed with other markers, such as cholecystokinin (CCK) or calretinin (CR) (Xu et al., 2010; Kubota et al., 2011; Cauli et al., 2014; Zeisel et al., 2015).

Little is known about the GABAergic neurons that co-express VIP with CCK since this group represents a small percentage of neurons in the cortex. Further studies using transgenic mice to identify these neurons are still needed to resolve their role in cortical physiology. The expression of different neuronal molecules and peptides within the same GABAergic neuron class might indicate that these neurons play multiple roles in cortical circuits.

## Excitatory and Inhibitory Synapses Onto Cortical VIP-Expressing Neurons

The anatomical presence of excitatory synapses (presynaptic boutons) onto cortical VIP-expressing neurons are preferentially studied from long-range projections originating in different brain areas. In this review, we consider “long-range” GABAergic projections when they connect brain areas associated with different sensory modalities and/or executive functions. For this reason, we do not consider lateral or interlaminar connectivity within one brain area and between subregions of a brain area as “long-range” GABAergic connectivity. Rather, we include projections that connect areas with different functions within the ipsilateral hemisphere, such as motor and somatosensory cortex, as well as projections that connect cortical regions with the same function *via* the corpus callous (interhemispheric projections).

Most studies on the presynaptic local connectivity between GABAergic and glutamatergic neurons onto VIP-expressing neurons are performed electrophysiologically. These studies unveil that, in layer 2/3 of the S1, the probability of connections between local excitatory neurons and the VIP-expressing neurons is very low (about 20%; Karnani et al., 2016b). These connections are also characterized as depressing short-term synaptic plasticity (Karnani et al., 2016b).

**FIGURE 1:**
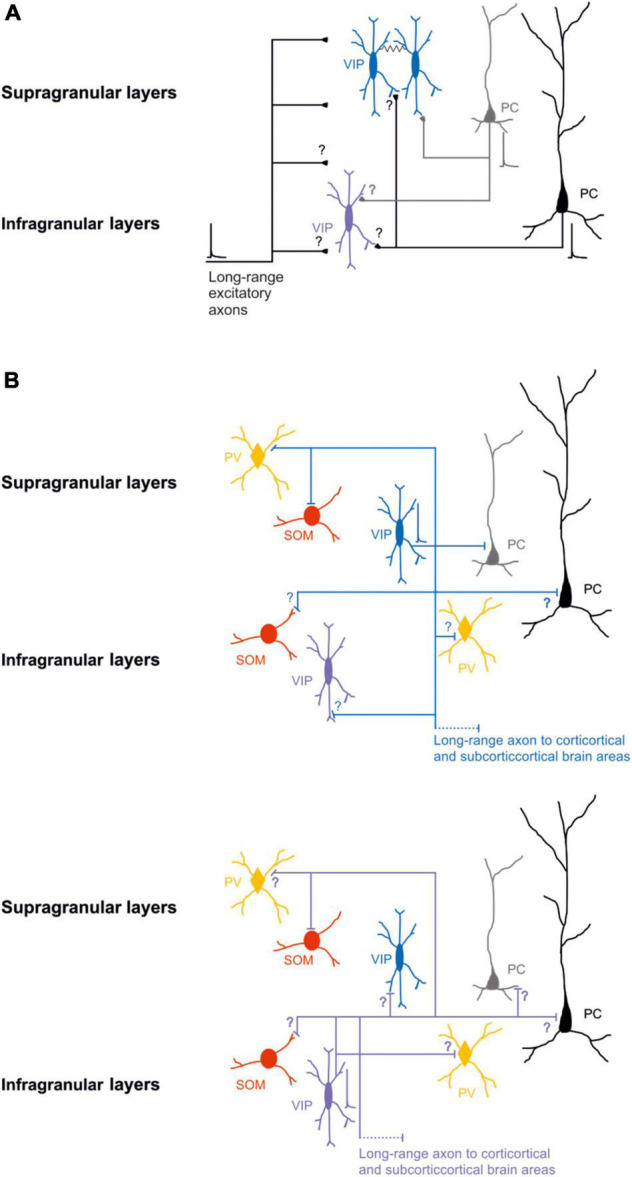
Schematic wiring diagrams of neuronal circuits in the supragranular and infragranular cortical layers in which VIP-expressing neurons are involved. **(A)** Synaptic excitatory inputs from local and distal brain areas onto superficial and deep VIP-expressing neurons. **(B)** Synaptic outputs from supragranular VIP-expressing neurons (top) and infragranular VIP-expressing neurons (bottom) onto other types of neurons. Note that most of the connectivity between the different classes of neurons is still not known. VIP-expressing neurons are also electrically connected black line. Blue, VIP supragranular layer and purple VIP infragranular layer; Red SOM; orange PV; and gray superficial excitatory neurons and black deep excitatory neurons. Thick lines represent the axons that carries information and thin lines represent axons that are not active. Schematic action potentials are present depending on which neuronal circuit motive is recruited.

Specifically, it has been shown that, in S1, layer 2/3 bipolar VIP-expressing neurons receive significantly more excitatory input from layer 2/3 and significantly less from the other layers (Xu and Callaway, 2009). After immunohistochemical labeling of presynaptic boutons and postsynaptic structures of the layer 2/3 VIP-expressing neurons of the mouse S1, Sohn et al. (2016) described that both cortico-cortical and thalamic excitatory synapses contact the distal dendrites of the VIP-expressing neurons (Figure 1A).

Moreover, Ji et al. (2016) demonstrated that in the mouse auditory (A1) and visual cortex (V1) cortex layer 4, VIP-expressing neurons are innervated by thalamic axons, and the synaptic strength was much weaker compared with excitatory and PV-expressing neurons.

Taken together, these studies revealed that synaptic inputs onto VIP-expressing neurons had preferences for specific layers and somato-dendritic compartments. In addition, the revealed layer 4 cell-type specificity connectivity of thalamocortical innervation suggests that VIP-expressing neurons are unlikely to contribute to feedforward inhibition. At the same time, they might be better suited to provide feedback inhibition onto pyramidal neurons (Kapfer et al., 2007; Silberberg and Markram, 2007; Ma et al., 2010; Adesnik et al., 2012; Xu et al., 2013).

GABAergic postsynaptic inputs onto VIP-expressing neurons received more attention (Staiger et al., 1997, 2002; Olah et al., 2007). VIP-expressing neurons show a low number of inhibitory synapses along their somato-dendritic arborization. Specifically, the synapses from PV-expressing neurons target the soma and the proximal dendrites of the VIP-expressing neurons. In contrast, the SOM-expressing neurons contact the VIP-expressing neurons’ distal apical and basal dendrites. Reciprocal boutons between VIP-expressing neurons are found in all somato-dendritic compartments but are pretty sparse (Figure 1B top; Sohn et al., 2016).

It has also been demonstrated that VIP-expressing neurons, that express choline acetyltransferase (VIP-ChAT), can co-release acetylcholine with GABA onto other GABAergic neurons, mainly in layer 1 and onto VIP-ChAT neurons. This suggests a possible microcircuit loop between these neurons that could be recruited within the subnetwork to amplify the incoming stream of information (Granger et al., 2020). In summary, at the microcircuit level, it has been suggested that VIP-expressing neurons receive different local and long-range glutamatergic/excitatory inputs (Figure 1A) and local synaptic inputs from other GABAergic neurons (Figure 1B top). It appears, at least in the superficial layer of the S1, that the density of excitatory input that arises from other cortical regions is larger compared to excitatory fibers from the thalamus, and the inhibitory synapses’ density onto VIP expressing is similar to the PV-expressing and SOM-expressing neurons (Sohn et al., 2016). This shows a non-selective inhibition of VIP from the other two subclasses of GABAergic neurons, but the main excitatory inputs arise from other cortical areas. This could form a neuronal motif in which cortical VIP-expressing neurons are a key player in processing the incoming inputs (see next paragraph).

## Local Circuit Organization of VIP-Expressing Neurons

In recent years, numerous studies have been focused on the role of the superficial layer VIP-expressing neurons (Pronneke et al., 2015; Zhou et al., 2017; Almasi et al., 2019), while fewer have assessed the role of the deep layers VIP-expressing neurons (Figure 1B bottom; Xu and Callaway, 2009; Pronneke et al., 2015) due to the lower density of these neurons (Yu et al., 2019). For this reason, the organization of the underlying circuit of deeper layer VIP-expressing neurons and their role in cortical processing remains elusive. While the small numbers of these neurons poses a challenge, the use of opsins and transgenic Cre-mouse lines permits to pinpointing the direct and indirect inputs to VIP-expressing neurons as a population (He et al., 2016).

The strategy of driving the activity of channelrhodopsin-expressing VIP-expressing neurons has mainly been used to identify and compare the synaptic strength output onto different classes of neurons in layer 2/3 of the S1 and the primary visual cortex (V1; Lee et al., 2013; Pfeffer et al., 2013; Neske and Connors, 2016; Garcia-Junco-Clemente et al., 2017; Krabbe et al., 2019). Mainly, VIP-expressing neurons inhibit Martinotti neurons that express somatostatin in both cortices (Pfeffer et al., 2013; Walker et al., 2016).

In addition, Walker et al. (2016), using a paired recording technique in the S1, demonstrated that VIP-expressing input onto Martinotti-SOM-expressing neurons facilitated at high frequency. In contrast, PV-expressing input resulted in frequency independent depression. This suggests that differences in the connection properties among these neurons enable disinhibition with distinct temporal features.

The preferential inhibition of the SOM-expressing neurons in the S1 (most likely Martinotti neurons) might regulate and narrow the lateral inhibition after sustained GABA release from SOM-expressing to nearby pyramidal neurons (Karnani et al., 2016a). *In vitro* studies have also shown that during spontaneous network cortical activity, such as up and down cortical states, the selective photo-activation of VIP-expressing neurons does not affect the layer 2/3 pyramidal neurons firing, highlighting the crucial role in the neuronal plasticity properties and the network state at which the circuit is engaged (Neske and Connors, 2016).

VIP-expressing neurons at the microcircuit level in different brain structures such as V1, A1, and S1 are synaptically connected to other classes of GABAergic and non-GABAergic neurons. However, all the previous studies aimed to determine the role of the VIP-expressing neurons in cortical processing reached the same conclusion as “the general VIP-expressing neurons canonical circuit organization.” These neurons are involved in a cortical disinhibitory (or inhibition of inhibition) motif (for review see: Kullander and Topolnik, 2021).

Notably, VIP-expressing neurons preferentially inhibit SOM-expressing neurons in superficial layers to break the inhibition on excitatory neurons (Lee et al., 2013; Pfeffer et al., 2013; Neske and Connors, 2016; Krabbe et al., 2019). An exciting aspect of the high connectivity found *in vitro* studies between VIP-expressing neurons and the SOM-expressing neurons is the inverse relationship of the number of neurons in the different layers. Mainly, the SOM-expressing neurons are present in deeper layers (Yavorska and Wehr, 2016), but VIP-expressing neurons are more present in the superficial layers (Pronneke et al., 2015, 2020). This intracortical connectivity, however, is still poorly studied (Figure 1B).

*In vivo* studies also have confirmed the functional role of the VIP→SOM disinhibitory motif. During selective photo-activation of the VIP-expressing neurons, the firing activity of the different neurons shifts to higher or lower rate/frequencies depending on the type of neurons (Pfeffer et al., 2013; Pi et al., 2013; Krabbe et al., 2019). More specifically, SOM-expressing neurons in the sensory and motor cortices are preferentially inhibited by photo-activation of VIP-expressing neurons, which leads to relief of excitatory neurons from suppression at least in the superficial layers of the cortex (Pfeffer et al., 2013; Pi et al., 2013; Garcia-Junco-Clemente et al., 2017; Keller et al., 2020).

In addition, it has been demonstrated that layer 2/3 pyramidal neurons innervate VIP- and SOM-expressing neurons with different inputs dynamics. Mainly, layer 2/3 pyramidal neurons in the sensory cortices formed depressing synapses onto VIP-expressing neurons and facilitating synapses onto SOM-expressing neurons (Karnani et al., 2016b). These results suggest that the VIP- and SOM-expressing neurons can be activated at different times during the excitation of the layer 2/3 circuits (Karnani et al., 2016b). Interestingly, Karnani et al. (2016b) also demonstrated that the VIP- and SOM-expressing neurons are excited by non-overlapping sets of nearby layer 2/3 pyramidal neurons. This indicates that VIP-expressing neurons are embedded in different local excitatory circuits (Karnani et al., 2016b), working as a single unit cooperating with other GABAergic neurons to control the firing selectively and specifically of pyramidal neurons (Hertag and Sprekeler, 2019).

One of the more exciting aspects is that none of the studies mentioned above took into consideration that the cortical and subcortical VIP-expressing neurons are highly electrically connected with each other and with different classes of GABAergic neurons (Figure 1A; Karnani et al., 2016b; Francavilla et al., 2018). This leaves the question of what role this type of local connectivity has in controlling the activity of excitatory neurons during the synchronous network activity (Lee et al., 2013; Pfeffer et al., 2013; Krabbe et al., 2019). It has been shown that the activity of a single VIP-expressing neuron, through the electrical synapses, amplifies and synchronizes the response to control a broader range of local and distal target neurons (Karnani et al., 2016a,b; Hertag and Sprekeler, 2019).

In the medial prefrontal cortex (mPFC), a subclass of VIP-expressing neurons that co-expresses the choline acetyltransferase (ChAT) excites both GABAergic and glutamatergic neurons in different cortical layers (Obermayer et al., 2019). Even though the probability of finding these connections is low, the photo-activation of this circuit plays an essential role at the microcircuit level for behaviorally relevant tasks. The VIP-ChAT-expressing neurons do not provide inhibitory and/or disinhibitory control over the pyramidal neurons but exert direct excitation onto pyramidal neurons, showing a different mechanism of how VIP-ChAT-expressing neurons can regulate the activity of pyramidal neurons (von Engelhardt et al., 2007; Obermayer et al., 2019).

Another source of regulating cortical circuits is that VIP-expressing neurons release the peptide VIP, stored at the presynaptic level, into dense-core vesicles (Whittaker, 1989). In an initial study, Goadsby and Shelley (1990) demonstrated that high-frequency stimulation of the facial nerve results in the cortical release of VIP in the anesthetized cat. The vasoactive intestinal polypeptide (VIP) is not only a cell-type-specific molecular marker but is also a 28 amino-acid neuropeptide that has been shown to work as a neurotransmitter, neurotrophic or neuroprotective factor in the peripheral and central nervous system (Borbely et al., 2013; Deng and Jin, 2017). It has been shown that VIP acts through the VPAC_1_ and VPAC_2_ receptors that are part of the Group II receptor (GPCR) family (for review see Cunha-Reis and Caulino-Rocha, 2020). Mainly, in the hippocampus, the activation of the VPAC_1_ receptors inhibits voltage-gated calcium channel-dependent GABA exocytosis through a G_*i/o*_ and PKA-independent, and partially PKC-dependent, mechanism (Cunha-Reis et al., 2017). On the other hand, VPAC_2_ receptors activation enhances voltage-gated calcium channel-dependent GABA exocytosis by a G_*s*_/PKA/PKC-dependent mechanism. This suggests an additional way of modulating neuronal circuits during specific brain oscillations and possibly with the further activation of non-neuronal cells (Ashur-Fabian et al., 1997).

## Functional Organization of the Neuronal Circuits Involving VIP-Expressing Neurons

Every brain structure does not work in isolation. Each neuronal class is wired so that the activation of that class alters the activity of specific downstream neurons in ways that we are only starting to understand. Nonetheless, the role of any particular type of neuron at the local and distal network during specific behavior remains still unclear.

In recent years, it has been possible to develop new tools for characterizing and modulating the activity at the somato-dendritic and axon levels of different classes of neurons. These methods have revealed how individual classes of GABAergic and glutamatergic neurons are engaged in processing, at the microcircuit level, the incoming information from distal brain areas. Anatomical studies have shown that the major cortico-cortical inputs along the somato-dendritic compartments of GABAergic neurons in all layers of the S1 are from the primary motor cortex (M1), the secondary somatosensory cortex (S2), and contralateral S1 (S1; Wall et al., 2016).

Although all the classes of GABAergic neurons receive similar long-range excitatory inputs, VIP-expressing neurons localized in the deeper layers receive a higher density of excitatory inputs than the other inhibitory neuron classes (Wall et al., 2016). Immunofluorescence studies have also shown that VIP-expressing neurons in superficial layers of S1 receive most of the long-range excitatory synapses from cortico-cortical and thalamocortical input onto their distal dendrites (Sohn et al., 2016). Other long-distance excitatory inputs, from subcortical brain structure to GABAergic neurons in the S1, are from the ventral posterior nucleus (VPM) and the posteromedial complex (POm) of the thalamus (Staiger et al., 1996; Sohn et al., 2016; Wall et al., 2016; Guet-McCreight et al., 2020; Figure 1A).

From a functional point of view, physiological and anatomical studies confirmed that optogenetic stimulation of terminals arising from the POm increases the firing activity of VIP- and PV-expressing neurons. This effect induces disinhibitory control at the microcircuit level of the pyramidal neurons through the inhibition of SOM-expressing neurons. Moreover, the selective inhibition of the S1 VIP-expressing neurons is sufficient to induce LTP in layer 2/3 pyramidal neurons when POm inputs are photo-activated at specific frequencies. This suggests that VIP-expressing neurons are the key player in the disinhibitory circuit (Audette et al., 2018; Williams and Holtmaat, 2019).

**FIGURE 2:**
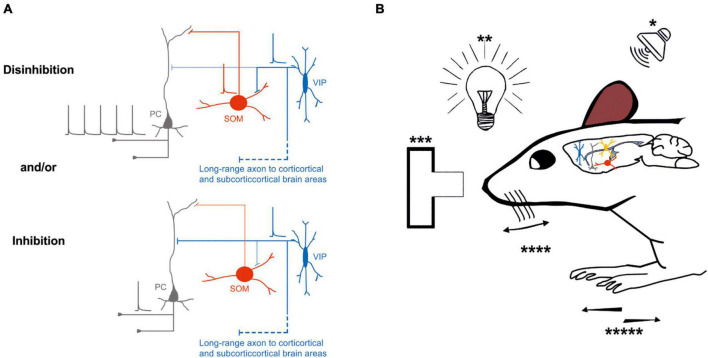
Possible inhibitory and/or disinhibitory circuits are entrained during specific behavioral tasks. **(A)** Top left, Schematic representation of the disinhibitory circuit, in which the activity of an excitatory neuron (gray) is increased by the inhibitory effect of VIP-expressing neuron (blue) onto SOM–expressing neuron inhibition (red); bottom left, schematic representation of direct inhibition of the excitatory neuron (gray) by VIP-expressing neuron (blue). **(B)** External stimuli such as auditory (*), visual (**), decision making (***), whisking (****), or running (*****) entrain different circuits motive: inhibition or disinhibition of the excitatory neurons through the VIP-SOM microcircuit. Thick lines represent the axons that carries information and thin lines represent axons that are not active. Schematic action potentials are present depending on which neuronal circuit motive is recruited.

Similar results have been demonstrated by Lee et al. (2019). In their study, the activity of VIP-expressing neurons increases in the open arms of an elevated plus maze in response to hippocampal inputs, leading to a disinhibitory effect onto layer 2/3 pyramidal neurons of the mouse prefrontal cortex. Thus, optogenetic inhibition of this subclass of neurons effectively controls how hippocampal input generates prefrontal representations, which drive avoidance behavior. Optogenetic activation of cortical VIP-expressing GABAergic neurons influences mouse behavior and sensory processing, depending on the cortical area and where the inputs originate (Figure 2B).

In addition, the selective activation of excitatory inputs from M1 or cingulate cortex mostly depolarize VIP-expressing neurons in superficial layers compared to the other classes of GABAergic neurons in S1 or V1, inducing a disinhibitory effect onto layer 2/3 pyramidal neurons through the SOM-expressing neurons (Lee et al., 2013; Zhang et al., 2014). Overall, these studies show a similar principle and possible connectivity pattern of the VIP-expressing neurons in the superficial layers of the cortex. However, they leave unanswered questions about the long-range inputs onto VIP-expressing neurons in the infragranular layers, specifically their connectivity patterns and if there is a similar disinhibitory motif in these layers (Figures 1A,B bottom). These questions are important because in other cortical areas, such as the auditory and visual cortex, long-range excitatory inputs from the thalamus (MGB and dLGN) exert only weak depolarization onto VIP-expressing neurons, and these inputs are restricted to layer 4 (Kloc and Maffei, 2014; Ji et al., 2016).

More recent studies have shown that, depending on the input area, the excitatory responses of the three classes of GABAergic neurons in the superficial layers is region-dependent, suggesting that the same local circuit processes the information differently depending on the upstream information. More specifically, only long-range excitatory inputs arising from M1 induce a disinhibitory effect onto layer 2/3 pyramidal neurons *via* the VIP-expressing neurons in the barrel cortex (Lee et al., 2013). On the other hand, other sources of long-range excitatory inputs are not selective to a specific class of GABAergic neurons, indicating a different role of the VIP-expressing neurons in processing the information stream in the superficial layers (Naskar et al., 2021). Even though they were found using *in vitro* preparation, these exciting results suggest that, at least in the S1 barrel cortex, inputs from different brain structures recruit a specific class of GABAergic neurons preferentially. This indicates that the information flow from other brain areas is elaborated by the receiving brain region, depending on the activation of the upstream cortical input.

Still, most studies focus on the activation of GABAergic neurons in superficial layers, but how these neurons in the deep layers contribute to processing the incoming inputs from different brain areas is quite poorly studied (Figure 1A; Larkum et al., 2018). Notably, cortical VIP-expressing neurons also receive cholinergic inputs. The activation of these inputs selectively controls the spike activity of these neurons, increasing the complexity of regulation at the microcircuit level of this subclass of neurons (Dudai et al., 2020).

Overall, the recruitment of specific classes of neurons, such as VIP-expressing neurons, by long-range inputs might be determining either an inhibitory and/or disinhibitory effect at the microcircuit level that depends on different factors (Figure 2A). It is still unknown whether VIP-expressing neurons preferentially target different subclasses of glutamatergic neurons (Feldmeyer, 2012; Harris and Shepherd, 2015). Remarkably, the activation of VIP-expressing neurons could route the information processing to a specific pathway by inhibiting one subclass of pyramidal neurons while providing disinhibition to another subclass of pyramidal neurons (Figure 2A).

It is very well-established that cortical neurons regulate the activity of neurons across brain areas through long-range glutamatergic/excitatory projections, whereas inhibition is mediated by local feedforward and feedback circuits (for review, see Tremblay et al., 2016). It is, however, known that long-range GABAergic neurons are important circuit elements in many brain areas, such as the spiny projection neurons in the striatum and the Purkinje neurons in the cerebellum. Although the existence of cortical long-range GABAergic neurons has been proven anatomically (Ito and Yoshida, 1966; Seress and Ribak, 1983; Toth and Freund, 1992; Toth et al., 1993; Freund and Buzsaki, 1996; for review, see Caputi et al., 2013; Tremblay et al., 2016), previous studies have primarily focused on the local cortical circuit organization of GABAergic interneurons (Buzsaki, 1984; Ali et al., 1999; Holmgren et al., 2003; Maccaferri and Lacaille, 2003; Pouille and Scanziani, 2004; Silberberg and Markram, 2007; Pouille et al., 2009, 2013; Stokes and Isaacson, 2010; Hayut et al., 2011; Pfeffer et al., 2013; Crandall and Connors, 2016), and inhibition is frequently described as being exclusively local.

However, more recent studies have shown that different classes of cortical GABAergic neurons project their axons to other cortices and subcortical brain areas, and their activation could influence the target region (Jinno and Kosaka, 2004; Tomioka et al., 2005; Higo et al., 2007, 2009; Tomioka and Rockland, 2007; McDonald et al., 2012; Melzer et al., 2012, 2017; Lee et al., 2014; Basu et al., 2016; Rock et al., 2016, 2018; Bertero et al., 2019, 2020, 2021; for review, see Tamamaki and Tomioka, 2010; Caputi et al., 2013; Melzer and Monyer, 2020). Specifically, for this review, VIP-expressing neurons in the auditory and motor cortex send long-range axons to different cortical and subcortical brain structures (Bertero et al., 2021), having the potential to exert their inhibition onto specific classes of neurons both locally and at the distal brain areas (Figures 1B, 2A). Further characterization of VIP-expressing neurons at the local microcircuit along all cortical layers is needed to unveil the role of this subclass of GABAergic neurons during specific brain oscillation (coherence or power) and in shaping the incoming and outgoing flow of the information stream. The recruitment of this class of neurons and how they elaborate the information is still an open question.

## Role of Cortical VIP-Expressing Neurons in Behaving Animals. Disinhibition vs. Inhibition?

A key question for neuroscientists is to correlate how each class of neurons can promote the transformation of the sensory signals into reward-learning and action-selection behaviors in the brain and how these mechanisms are altered in pathological conditions.

Nowadays, the possibility to test the different hypotheses in behaving animals using an enormous number of different tools to identify and control specific classes of neurons is helping the neuroscience community to uncover and understand the contribution of each neuronal class during specific tasks in awake behaving animals.

So far, most of the studies have focused their attention on the role of PV- and SOM-expressing neurons in different brain structures during certain behavioral paradigms to link the behavioral results with the local connectivity that generates that behavior. Selective mouse transgenic Cre-lines are widely used to correlate the activity of GABAergic neurons by cell-type during cortical processing. Specifically, a well-characterized VIP-Cre line is currently used to study the role of VIP-expressing neurons in cortical information processing during relevant behavioral tasks (Pronneke et al., 2015). However, understanding the behaviorally relevant increase/decrease of activity in the cortical VIP-expressing neurons is still at the early stages of investigation.

To address the relevance of VIP-expressing neuron activity fluctuations, experimenters tried different behavioral conditions with optogenetic manipulations to control their activity (Yavorska and Wehr, 2016; Bigelow et al., 2019; Krabbe et al., 2019). Similar approaches were used in other rodents’ brain areas, such as the A1 and the V1 cortex. These studies have shown that during animal movement, the VIP-expressing neurons are highly active, generating an overall disinhibitory effect onto local pyramidal neurons (Pi et al., 2013; Fu et al., 2014; Bigelow et al., 2019; Keller et al., 2020; Yavorska and Wehr, 2021; Figure 2).

Specifically, in both the primary auditory and visual cortex, the movement of the mouse affects both the local circuit activity and the information processing. Particularly, selective activation of VIP-expressing neurons in the A1 enhances only the local circuit activity during the auditory stimuli but not the information processing (Bigelow et al., 2019; Figure 2B). Interestingly, Yavorska and Wehr (2021) demonstrated how animal running behavior and optogenetic activation of VIP-expressing neurons differentially modulate sound-evoked activity across layers in the mouse auditory cortex. Mainly, they found that running increased spontaneous firing rates but decreased evoked firing rates, resulting in the reduced neuronal encoding of sound in all cortical layers. In contrast, the photo-activation of VIP-expressing neurons increased spontaneous and evoked firing rates and did not affect sound encoding. Overall, Yavorska and Wehr’s (2021) results indicated that the VIP-expressing neurons circuits do not mediate the effects of locomotion in the auditory cortex (Figure 2B).

In addition, Pi et al. (2013) measured neurons’ activity in the auditory cortex during a specific task in which a mouse was rewarded or punished depending on the tone presented. Remarkably, VIP-expressing neurons were strongly activated by reward and punishment signals (Pi et al., 2013). However, the strong recruitment of the VIP-expressing neurons did not correlate with an increased spiking of other nearby neurons, as can be expected by the identified canonical cortical disinhibitory circuit motif (for review, see Pfeffer, 2014; Figure 2A). One other important aspect of this disinhibitory circuit is that the VIP-expressing neurons strongly inhibit the SOM-expressing neurons, which in turn preferentially inhibit the pyramidal neurons’ distal dendrites (Somogyi et al., 1998). This distal inhibition can open a window to increase synaptic integration or plasticity along the distal dendrites of the pyramidal neurons.

Are the long-range cortical VIP-expressing neurons (Bertero et al., 2021) part of a long-range disinhibitory circuit leading to integration and plasticity across cortical and subcortical areas? Future experiments will need to address these open questions on the role of the long-range VIP-expressing neurons that can dynamically affect the cortico-cortical processing (Figure 2A).

In the visual cortex, VIP-expressing neurons increase their firing rate mainly when the background and the stimulus are different. This increase in the firing rate reduces the activity of SOM-expressing neurons generating a disinhibitory effect onto local pyramidal neurons (Keller et al., 2020; Figure 2A). Moreover, during locomotion, the activity of VIP-expressing neurons is increased, and this effect is independent of the visual stimulus. However, this activation arises from nicotinic inputs from the nucleus of the diagonal bans of Broca, a cholinergic center in the basal forebrain (Fu et al., 2014; Figure 2B).

Interestingly, as shown by Fu et al. (2014), VIP-expressing neurons are more active in V1 during locomotion than other sensory cortices. The VIP-expressing neurons in the visual cortex are also the target of top-down inputs and mediate enhancement of local pyramidal cell activity in response to activation of those inputs (Zhang et al., 2014). It has been demonstrated that the mouse V1 is necessary to detect low contrast visual stimuli (Glickfeld et al., 2013). Selective optogenetic activation of either PV-, SOM-, or VIP-expressing of GABAergic neurons has a different effect on the contrast-detection observed in the mouse V1. The activation of VIP-expressing neurons lowers the thresholds of contrast detection while the activation of SOM- or PV-expressing neurons raises it (Cone et al., 2019). This suggests that the perception of low contrast stimuli is strongly enhanced by VIP neuron activity in V1.

Recently, Millman et al. (2020) have investigated the influence of stimulus contrast and locomotion on the visual responses of VIP-, SOM-expressing neurons, and pyramidal neurons in mouse V1. Particularly, they found that the SOM- and VIP-expressing neurons differentially responded to high and low contrast. Mainly, the SOM-expressing neurons responded exclusively at low contrast that is congruent with self-motion during locomotion. In this study, Millman et al. (2020) demonstrated that the VIP-driven disinhibition at low contrast could strongly increase the activity of pyramidal neurons, even though both pyramidal and SOM-expressing neurons are characterized by a lower activity during low contrast regime. Overall, the Millman et al. (2020) results indicated that the VIP-expressing neurons, *via* the VIP-disinhibitory circuit motif, can amplify the response of the pyramidal neurons to weak but behaviorally relevant stimuli such as low contrast front-to-back motion that can be fundamental for the detection of approaching obstacles during locomotion (Figure 2B).

It has also been demonstrated that the context can modulate the response of specific visual stimuli in the visual cortex. Lately, Keller et al. (2020) have identified a canonical VIP-driven disinhibitory circuit that, by inhibiting the SOM-expressing neurons, modulates the response in the mouse V1 accordingly to the similarity between the surround and the stimulus (Figure 2B).

In another sensory cortex, such as the S1 barrel cortex, it has been shown that layer 2/3 VIP-expressing neurons increase the firing rate during active whisking by disinhibiting the pyramidal neurons (Lee et al., 2013; Figures 2A,B). In addition, multiple studies have characterized different sub-classes of GABAergic neurons engaged during whisker-dependent behavior (Sachidhanandam et al., 2016; Yu et al., 2019). In their study, Sachidhanandam et al. (2016) trained mice to obtain a water reward in response to whisker deflection while recording the activity from genetically targeted GABAergic neurons in layer 2/3 of the mouse barrel cortex. They found that the SOM-expressing neurons fired at low rates, while both PV- and VIP-expressing neurons fired at high rates during whisker stimulation. Moreover, Yu et al. (2019) demonstrated that the VIP-expressing neurons during active tactile behavior are activated by non-sensory inputs that disinhibit both pyramidal and PV-expressing neurons. Overall, these studies indicate that the different class of GABAergic neurons is characterized by a specific firing dynamic that depends on the behavioral task execution (Sachidhanandam et al., 2016; Yu et al., 2019).

It has also been demonstrated that a subpopulation of VIP/ChAT positive neurons in the superficial layers are recruited during whisker movements and play a key role in encoding the external stimuli (Dudai et al., 2020). At this point, it is legitimate to ask: Is the activity of VIP-expressing neurons during locomotion applied only to sensory cortices?

The VIP-expressing neurons are also involved in motor skill learning (Adler et al., 2019). In their study, Adler et al. (2019) trained mice to run forward or backward at fixed speeds on a treadmill. During this motor learning task, GABAergic and pyramidal neurons exhibited different activation patterns. The layers 2/3 pyramidal neurons were characterized by sequential activation patterns (Adler et al., 2019), while the SOM-expressing neurons exhibited diverse responses. Their activity was enhanced, suppressed, or unchanged at the onset of forward and backward running, and their activation suppressed the sequential activation of pyramidal neurons. In addition, Adler et al. (2019) also revealed that VIP-expressing neurons could inhibit SOM-expressing GABAergic neurons, which leads to relief of pyramidal neurons from suppression (VIP-disinhibitory motif) permitting synaptic plasticity and learning to take place (Figures 2A,B).

Another study focused on the role of the VIP-expressing neurons in behavioral performance and neuronal action plan in the dorsomedial prefrontal cortex (Kamigaki and Dan, 2017). When VIP-expressing neurons were selectively photo-activated, through the disinhibitory circuit, performance on the go/no-go task was enhanced (Figure 2). This study highlights the role of this class of neurons in modulating behavioral performances and tasks.

Furthermore, medial prefrontal cortical inputs arising from the ventral hippocampus activate VIP-expressing neurons while animals are in the open arm of the elevated plus-maze. This has an overall disinhibitory effect at the prefrontal cortex microcircuit. Additionally, selective inactivation of VIP neurons during this task elicits an increase in avoidance behavior (Lee et al., 2019; Figures 2A,B). Overall, it is still intriguing to speculate about the role of layer 5 VIP-expressing neurons in cortical processing: are they establishing a disinhibitory local circuit motif (VIP→SOM→L5 Pyramidal neurons) mirroring their main organization in the layer 2/3 of the cortex? It is also possible to speculate that the layer 5 VIP-expressing neurons directly inhibit pyramidal neurons (VIP→L5 Pyramidal neurons).

Therefore, we think that the role of the layer 5 VIP-disinhibitory and VIP-inhibitory circuit motif still needs further elucidation (see Figures 1B bottom, 2A).

## The Role of VIP-Expressing Neurons in Cortical Dysfunction

Alterations of GABAergic neurons, at the molecular and circuit level, have been proven to induce a dysfunctional effect leading to several diseases such as epilepsy, autism, and anxiety (Yu et al., 2006; Marin, 2012; Lewis, 2014). Even though the GABAergic neurons represent only a small fraction of the entire cortical neuronal population, selective mutation of specific genes in diverse GABAergic neurons might induce several pathological conditions.

VIP-expressing neurons release the peptide VIP activating neuronal and non-neuronal cells (see Section “Local Circuit Organization of VIP-Expressing Neurons” above). The asymmetric activation of the two receptors (VPAC1 and VPAC2) suggests that different signaling pathways regulate neuronal circuits’ activity. The alteration of VIP release and/or the VIP binding receptors could be potentially implicated in multiple pathophysiological conditions. It has been shown that the VIP peptide has a significant role in regulating neuronal activity, and the selective loss of this peptide is associated with several pathological disorders such as depression (Ivanova et al., 2012), Parkinson’s disease, and epilepsy (Hill, 2007; White et al., 2010). Indeed, in human temporal lobe epilepsy brains, the receptors for the VIP peptide are increased in expression in the focus of the seizure. In the animal model of temporal lobe epilepsy, although there is a severe reduction of particular types of neurons, the number of VIP-expressing neurons seems not to be affected (King and LaMotte, 1988). VIP knock-out (KO) mice exhibit alterations in memory tasks and social behavior (Stack et al., 2008). However, the role of the VIP-expressing neurons at the neuronal level and their role in pathological conditions still need to be elucidated.

Al the circuit level, only recently, several groups have found that activity of dysfunctional VIP-expressing neurons is associated with neuronal disorders. Alteration of the specific gene or gene expression regulators, which control the transcription factors, specifically in VIP-expressing neurons, are implicated in pathophysiological conditions. These effects could alter the inhibitory/disinhibitory impact of the expressing neurons at the micro and long-range circuit level and alter the animal’s behavior.

In the condition in which the gene ErbB4 that expresses the membrane-bound tyrosine kinase receptor is deleted, the V1 cortical circuits and neuronal synchrony between spike activity and brain oscillations in the gamma bands (40–60 Hz) are dysregulated, inducing an impairment in the information processing performed by neuronal circuits. This mutation reduces the activity of VIP-expressing neurons also witnessed with an increase excitatory neuronal activity, suggesting a direct inhibitory effect of the VIP (Batista-Brito et al., 2017). Other studies have found that the ablation of the gene that encodes for a gene expression regulator selectively reduced the cell vitality in adulthood and impaired intrinsic properties of VIP-expressing neurons. This lead to an increase in inhibitory inputs onto pyramidal neurons, indicating that a disinhibitory circuit is involved, and dysfunctional cortical oscillations. These effects induced a better spatial working memory and motor coordination in these mice but reduced their life span (Qiu et al., 2020).

In a complementary condition, the S1 VIP-expressing neurons lost their preferential abundance in the superficial layers. Moreover, the long-range inputs onto VIP-expressing neurons from ipsilateral and contralateral cortices were compromised, contributing to a dysfunctional elaboration of the incoming inputs (Hafner et al., 2021). In addition, Goff and Goldberg (2019) have shown that the neocortical VIP-expressing neurons in Scn1a^+/–^ mice, a mouse model of Dravet syndrome, exhibited impaired excitability. Despite these two latest studies, it is still not known whether there is an inhibitory/disinhibitory alteration due to the mutation of the Scn1a gene.

Overall, these studies highlight how developmental dysregulation, gene expression ablation, and structural abnormalities specifically in VIP would lead to pathophysiological conditions, highlighting the crucial role of VIP-expressing, GABAergic neurons in neuronal circuit dysfunctions and neurological disorders.

## Conclusion and Perspectives

In recent years, with the advanced technological tools, it has been possible to selectively control specific groups of neurons, including the VIP-expressing neurons, which represent a small percentage of the entire GABAergic population. This has allowed great strides in understanding the physiological role of a particular type of neurons, from the detailed anatomy of the neurons up to their activity during specific tasks.

The VIP-expressing neurons are mainly present in the superficial layers of the cortex (Pronneke et al., 2015, 2020; Zhou et al., 2017; Almasi et al., 2019; Badrinarayanan et al., 2021), where most of the studies are performed but leaving almost unexplored the physiology of VIP-expressing neurons in deeper cortical layers. The difficulty of *in vivo* studies for the infragranular VIP-expressing neurons is their low number (Yu et al., 2019). So far, only *in vitro* studies elucidate anatomical and electrophysiological differences between superficial and deep cortical VIP-expressing neurons. They mainly confirm some dissimilarity about these neurons and suggest a potential differential role at the circuit level and most likely during behavior.

*In vitro* and *in vivo* studies have previously revealed a preferential inhibition of the VIP-expressing neuron to SOM-expressing neurons labeling them as the main players of the disinhibitory role at the local circuit level (Lee et al., 2013; Pfeffer et al., 2013; Pi et al., 2013). However, recent studies have suggested that this subclass of GABAergic neurons can inhibit and disinhibit excitatory neurons (Garcia-Junco-Clemente et al., 2017; Zhou et al., 2017; see for review Kullander and Topolnik, 2021).

Further studies *in vitro* and *in vivo* done in different brain areas and during various behavioral tasks are still needed to resolve the functional role of the VIP-expressing GABAergic neurons. The peculiar local and distal circuit in which these neurons are embedded suggests a more complex function without limiting their role in the disinhibitory effect onto pyramidal neurons by inhibiting the SOM-expressing neurons (Naskar et al., 2021).

One example of an additional function of VIP-expressing neurons is their ability to inhibit other GABAergic and non-GABAergic neurons (Pfeffer et al., 2013), leading to a different scenario at the circuit level. Additionally, VIP-expressing neurons of the mouse auditory and motor cortices can also send long-range projections to cortical and subcortical areas. Particularly, VIP-expressing neurons of the auditory cortex can reach the contralateral auditory cortex and the ipsilateral striatum and amygdala, as shown for Somatostatin- and Parvalbumin-expressing long-range neurons. Moreover, these neurons also send long-range GABAergic projections to the medial geniculate body and both superior and inferior colliculus. In addition, Bertero et al. (2021; Figures 1B, 2A) demonstrated that VIP-expressing neurons of the motor cortex also send long-range GABAergic projections to the dorsal striatum and contralateral cortex, suggesting that the long-range VIP projection is likely a general feature of the neocortex’s network. Even though these studies suggest an unforeseen role of this subclass of neurons, more physiological data is still needed to frame and better understand their role in cortical circuits and behavior.

Another characteristic, which could shape the role of these neurons and give a better definition to their inhibitory/disinhibitory effect, is their synaptic plasticity. The dynamics in which these neurons release their neurotransmitters influence cortical circuits (Cirelli and Tononi, 2000). So far, *in vitro* experiments in the superficial layer of the S1 barrel showed short-term facilitation of these synapses onto SOM-expressing neurons but a short-term depression onto PV-expressing neurons (Walker et al., 2016). Nevertheless, data in deep neocortical layers and *in vivo* are still lacking. Different groups tested the long-distance cortico-cortical and cortico-thalamic excitatory inputs onto VIP-expressing neurons stimulating axon terminals in the recipient structure. Lee et al. (2013) found that M1 excitatory axons showed synaptic depression after paired-pulse stimuli. However, most of the protocols used were a single photo pulse, leaving the aspect of short-term dynamics still unexplored. Another crucial aspect that is still missing in the puzzle of the neocortical role of the VIP-expressing neurons is during brain oscillations. These results on temporal dynamics are still missing due to their low percentage of neurons, especially in the deep neocortical layers.

The present review shows the complexity of the neocortical VIP-expressing neurons’ anatomical and electrophysiological properties and neurotransmitter release machinery. In addition, the characteristic input/output of the VIP-expressing neurons makes their collocation in the neocortical organization and function even more intriguing to resolve.

Many questions still need to be addressed concerning VIP-expressing neurons circuits in the neocortex and other brain areas. What role do the other subclasses of GABAergic neurons play in the disinhibitory circuit provided by the activation of VIP-expressing neurons? Do VIP-expressing neurons exert an inhibitory/disinhibitory effect in deeper neocortical layers? What is the role of this intracortical inhibition in the sensory integration of top-down and bottom-up information? What is the role of these neurons during specific neocortical activity in awake animals? Future experiments will provide further insight into the complexity of the local circuit’s organization of the VIP-expressing neurons and their role in neocortical processing (for review see Larkum, 2013).

Overall, this review has highlighted the differential roles of the VIP-expressing neuron in cortical processing. However, future experiments are still necessary to determine the possible VIP-expressing neurons inhibitory and/or disinhibitory motifs across cortical layers and different brain structures.

## Author Contributions

AjA and IM wrote and commented on the manuscript. Both authors contributed to the article and approved the submitted version.

## Conflict of Interest

The authors declare that the research was conducted in the absence of any commercial or financial relationships that could be construed as a potential conflict of interest.

## Publisher’s Note

All claims expressed in this article are solely those of the authors and do not necessarily represent those of their affiliated organizations, or those of the publisher, the editors and the reviewers. Any product that may be evaluated in this article, or claim that may be made by its manufacturer, is not guaranteed or endorsed by the publisher.
